# Analysis of Methods of Suicide Among US Military Veterans Recently Separated From Military Service

**DOI:** 10.1001/jamanetworkopen.2022.10731

**Published:** 2022-05-06

**Authors:** Ian H. Stanley, Chandru Ravindran, Sybil W. Morley, Brady M. Stephens, Mark A. Reger

**Affiliations:** 1 National Center for PTSD, VA Boston Healthcare System, Boston, Massachusetts; 2 Department of Psychiatry, Boston University School of Medicine, Boston, Massachusetts; 3 Veterans Integrated Service Network (VISN) 2, Center of Excellence for Suicide Prevention, Canandaigua, New York; 4 Veterans Affairs Puget Sound Health Care System, Seattle, Washington; 5 Department of Psychiatry and Behavioral Sciences, University of Washington, Seattle

## Abstract

This cohort study compares firearm, suffocation, and poisoning suicide rates among recently separated veterans with those among the general veteran population and examines demographic and military characteristics associated with risk of method-specific suicide mortality among recently separated veterans.

## Introduction

Veterans who recently separated from military service have high suicide rates for several years after the transition.^1^ It is unknown whether this risk differs by method of suicide. This study aimed to examine (1) firearm, suffocation, and poisoning suicide rates among recently separated veterans (≤5 years) and the general veteran population and (2) demographic and military characteristics associated with risk of method-specific suicide mortality among recently separated veterans.

## Methods

This retrospective, population-based cohort study used data from the Veterans Affairs (VA)/Department of Defense Identity Repository and Mortality Data Repository.^2^ Because analyses were conducted as part of ongoing Veterans Health Administration operations and program evaluation conducted by the VA Office of Mental Health and Suicide Prevention, institutional review board approval and informed consent were not required per VA policy. We followed the Strengthening the Reporting of Observational Studies in Epidemiology (STROBE) reporting guideline.

Individuals who served active duty in the US Army, Navy, Air Force, or Marine Corps after September 11, 2001, and were separated from the Active Component or Selected Reserve between January 1, 2010, and December 31, 2019, were included in this recently separated cohort. Separation refers to complete separation from military status (discharge into civilian life) or transition from active status to a Reserve component category other than Selected Reserve (eg, Inactive Ready Reserve). Service members entered the cohort on their separation date and exited after 5 years (1825 days), on their date of death, or on December 31, 2019, whichever came first. The time at risk was calculated as the number of days from separation to exiting the cohort.

Race and ethnicity (American Indian or Alaskan Native, Asian, Black or African American, Hispanic, Native Hawaiian, Pacific Islander, White, and unknown) were determined from data in the repository. Race and ethnicity were assessed in this study because previous research has shown between-group differences in suicide risk.

We compared suicide mortality within 5 years of separation in the recently separated cohort with the overall suicide mortality of the general veteran population (regardless of separation date) between 2010 and 2019 using age-standardized mortality ratios (SMRs) and associated Poisson 95% CIs. Directly age-standardized rate ratios (RRs) and 95% CIs were generated for comparisons within the cohort between strata. For service members with multiple transitions, we used the characteristics associated with their latest separation. Missing sociodemographic data were addressed through pairwise deletion. Analyses were conducted from April 20, 2021, to September 27, 2021. Data were analyzed with SAS statistical software version 8.2 (SAS Institute).

## Results

The cohort included 2 323 692 recently separated veterans (1 943 755 men [83.6%]; 379 931 women [16.4%]; mean [SD] age at separation, 30.7 [9.9] years). Overall, 3573 suicides were identified within 5 years of military separation. Compared with suicide mortality in the general veteran population, recently separated veterans were at increased risk of suicide by any method (SMR, 1.04; 95% CI, 1.01-1.08) and firearms (SMR, 1.10; 95% CI, 1.06-1.15) (Table 1). Sex-stratified analyses revealed that the increased risk for firearm suicide among recently separated veterans was specific to male veterans (SMR, 1.11; 95% CI, 1.06-1.15). Among recently separated veterans, the risk of firearm suicide was elevated for those who were male, White, non-Hispanic, unmarried, last served in the Active Component, and last served in the Army or Marine Corps, compared with their respective demographic groups (Table 2).

**Table 1.** SMRs Comparing Risk of Suicide Within 5 Years for Recently Separated Veterans vs Risk of Suicide for the General Veteran Population (2010-2019), by Sex

Group	Suicides^a^											
	All			Firearm			Suffocation			Poisoning		
	Observed	Expected	SMR (95% CI)^b^	Observed	Expected	SMR (95% CI)^b^	Observed	Expected	SMR (95% CI)^b^	Observed	Expected	SMR (95% CI)^b^
All	3573	3430	1.04 (1.01-1.08)	2366	2141	1.10 (1.06-1.15)	794	806	0.99 (0.92-1.06)	259	299	0.87 (0.76-0.98)
Male	3356	3211	1.05 (1.01-1.08)	2259	2040	1.11 (1.06-1.15)	733	752	0.97 (0.91-1.05)	219	245	0.89 (0.78-1.02)
Female	217	239	0.91 (0.79-1.04)	107	109	0.98 (0.80-1.18)	61	60	1.01 (0.77-1.30)	40	56	0.71 (0.51-0.97)

Abbreviation: SMR, standardized mortality ratio.

^a^ We used *International Statistical Classification of Diseases and Related Health Problems, Tenth Revision* codes X60 to X84, Y87.0, and U03 to identify deaths for which suicide was listed as the underlying cause of death; firearm suicides were identified with codes X72 to X74, poisoning suicides with codes X60 to X69, and suffocation suicides with code X70.

^b^ Data are age adjusted.

**Table 2.** Age-Standardized RRs of Suicide Among Veterans Within 5 Years of Separation From Service, by Method

Group	Person-years	Suicides											
		All			Firearm			Suffocation			Poisoning		
		Suicides, No.	Rate^a^	RR (95% CI)^b^	Suicides, No.	Rate^a^	RR (95% CI)^b^	Suicides, No.	Rate^a^	RR (95% CI)^b^	Suicides, No.	Rate^a^	RR (95% CI)^b^
Sex													
Female	1 443 405	217	15.03	1 [Reference]	107	7.41	1 [Reference]	61	4.23	1 [Reference]	40	2.77	1 [Reference]
Male	7 456 674	3356	45.01	3.09 (2.69-3.54)	2259	30.30	4.25 (3.50-5.16)	733	9.83	2.41 (1.86-3.13)	219	2.94	1.05 (0.75-1.48)
Age, y^c^													
17-29	5 292 876	2604	49.20	NA	1732	32.72	NA	609	11.51	NA	157	2.97	NA
30-39	1 687 858	617	36.56	NA	400	23.70	NA	119	7.05	NA	63	3.73	NA
≥40	1 919 275	352	18.34	NA	234	12.19	NA	66	3.44	NA	39	2.03	NA
Race													
Black or African American	1 413 427	377	26.67	0.65 (0.59-0.73)	235	16.63	0.60 (0.53-0.69)	81	5.73	0.65 (0.52-0.82)	30	2.12	0.71 (0.48-1.05)
White	6 452 731	2782	43.11	1 [Reference]	1876	29.07	1 [Reference]	613	9.50	1 [Reference]	196	3.04	1 [Reference]
Other^d^	657 618	277	42.12	0.97 (0.85-1.09)	163	24.79	0.85 (0.72-0.99)	72	10.95	1.14 (0.90-1.46)	25	3.80	NA^e^
Ethnicity													
Hispanic	944 167	288	30.50	0.71 (0.63-0.80)	181	19.17	0.67 (0.57-0.78)	74	7.84	0.83 (0.66-1.06)	23	2.44	0.81 (0.52-1.24)
Non-Hispanic	7 896 477	3271	41.42	1 [Reference]	2175	27.54	1 [Reference]	718	9.09	1 [Reference]	234	2.96	1 [Reference]
Branch													
Air Force	1 557 126	427	27.42	1 [Reference]	280	17.98	1 [Reference]	87	5.59	1 [Reference]	35	2.25	1 [Reference]
Army	4 454 763	1906	42.79	1.38 (1.24-1.54)	1248	28.01	1.40 (1.23-1.60)	443	9.94	1.51 (1.19-1.91)	140	3.14	1.25 (0.85-1.84)
Marines	1 348 092	769	57.04	1.59 (1.40-1.80)	553	41.02	1.77 (1.52-2.07)	148	10.98	1.37 (1.03-1.82)	44	3.26	1.32 (0.81-2.15)
Navy	1 540 128	471	30.58	0.98 (0.86-1.12)	285	18.50	0.92 (0.78-1.09)	116	7.53	1.13 (0.85-1.50)	40	2.60	1.04 (0.65-1.66)
Component													
Active	5 987 333	2643	44.14	1.25 (1.16-1.35)	1769	29.55	1.31 (1.19-1.44)	569	9.50	1.07 (0.91-1.25)	191	3.19	1.34 (1.01-1.78)
Reserve	2 912 776	930	31.93	1 [Reference]	597	20.50	1 [Reference]	225	7.72	1 [Reference]	68	2.33	1 [Reference]
Education level^c^													
Non–high school graduate	1 238 122	649	52.42	2.39 (1.93-2.98)	406	32.79	2.29 (1.75-2.99)	160	12.92	2.62 (1.63-4.21)	58	4.68	3.02 (1.48-6.15)
High school graduate	6 367 649	2686	42.18	1.80 (1.46-2.21)	1808	28.39	1.84 (1.43-2.38)	584	9.17	1.70 (1.08-2.68)	176	2.76	1.77 (0.90-3.50)
Higher degree	1 209 730	217	17.94	1 [Reference]	140	11.57	1 [Reference]	44	3.64	1 [Reference]	22	1.82	1 [Reference]
Marital status^c^													
Married	2 083 707	487	23.37	1 [Reference]	299	14.35	1 [Reference]	111	5.33	1 [Reference]	52	2.50	1 [Reference]
Not married	6 631 525	3030	45.69	1.24 (1.08-1.43)	2037	30.72	1.45 (1.21-1.75)	666	10.04	0.86 (0.66-1.13)	199	3.00	1.34 (0.85-2.12)

Abbreviations: NA, not applicable; RR, rate ratio.

^a^ Rate is the crude rate per 100 000 population.

^b^ Data are age adjusted.

^c^ Refers to status at separation.

^d^ Other race includes the following categories: (1) Asian, Native Hawaiian, or Pacific Islander; (2) American Indian or Alaskan Native; and (3) unknown.

^e^ Data are suppressed because of 0 counts in strata-specific cells.

## Discussion

In this cohort study, we found that recently separated male veterans were at increased risk for firearm suicide compared with the general veteran population, adjusting for age. We speculate that recently separated veterans may have more proximal familiarity and comfort with firearms and/or are more likely to own or have access to firearms, thereby increasing their risk for firearm suicide.^3^ Mechanisms and processes accounting for elevated risk for firearm suicide among recently separated veterans requires additional inquiry. A limitation of this study is that we ascertained suicide deaths via death certificates, which are subject to misclassification.

These findings have potential clinical and programmatic implications to prevent suicide among recently separated veterans. One approach is lethal means safety counseling (LMSC). The White House recently called on the VA and Department of Defense, among other federal agencies, to create an interagency action plan to broadly implement LMSC.^4^ This study suggests that LMSC and other public health efforts that promote safe firearm storage practices might be especially important for recently separated veterans.^5,6^
